# Supplementary material for: Development of chemokine network inhibitors using combinatorial saturation mutagenesis
Source: Commun Biol. 2025 Apr 3;8:549. doi: 10.1038/s42003-025-07778-6 (PMC11969024; doi:10.1038/s42003-025-07778-6)
Supplement: Supplementary file 4 — Supplementary Data 2 [file 42003_2025_7778_MOESM4_ESM.zip › SupplementaryData3/scripts/xNNKi.tar!/./script/analysis/JK/heatmaps/HD2Xselected_heatmap.pdf]

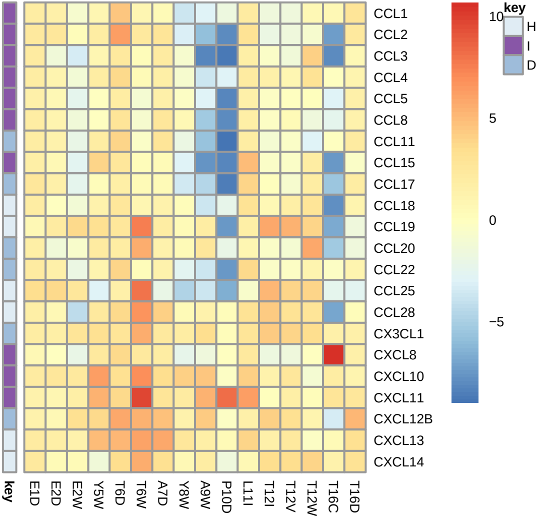

CCL1
CCL2
CCL3
CCL4
CCL5
CCL8
CCL11
CCL15
CCL17
CCL18
CCL19
CCL20
CCL22
CCL25
CCL28
CX3CL1
CXCL8
CXCL10
CXCL11
CXCL12B
CXCL13
CXCL14
key
E1D
E2D
E2W
Y5W
T6D
T6W
A7D
Y8W
A9W
P10D
L11I
T12I
T12V
T12W
T16C
T16D
10
5
0
−5
key
H
I
D
